# *In vivo* analysis of internal ribosome entry at the *Hairless* locus by genome engineering in *Drosophila*

**DOI:** 10.1038/srep34881

**Published:** 2016-10-07

**Authors:** Thomas K. Smylla, Anette Preiss, Dieter Maier

**Affiliations:** 1 Universität Hohenheim, Institut für Genetik (240), Garbenstr. 30, 70599 Stuttgart, Germany

## Abstract

Cell communication in metazoans requires the highly conserved Notch signaling pathway, which is subjected to strict regulation of both activation and silencing. In *Drosophila melanogaster*, silencing involves the assembly of a repressor complex by Hairless (H) on Notch target gene promoters. We previously found an in-frame internal ribosome entry site in the full length *H* transcript resulting in two H protein isoforms (H^p120^ and H^p150^). Hence, H may repress Notch signalling activity in situations where cap-dependent translation is inhibited. Here we demonstrate the *in vivo* importance of both H isoforms for proper fly development. To this end, we replaced the endogenous *H* locus by constructs specifically affecting translation of either H^p150^ or H^p120^ isoforms using genome engineering. Our findings indicate the functional relevance of both H proteins. Based on bristle phenotypes, the predominant isoform H^p150^ appears to be of particular importance. In contrast, growth regulation and venation of the wing require the concomitant activity of both isoforms. Finally, the IRES dependent production of H^p120^ during mitosis was verified *in vivo*. Together our data confirm IRES mediated translation of H protein *in vivo*, supporting strict regulation of Notch in different cellular settings.

Development and cellular differentiation of higher animals relies on cell-cell communication, involving a small number of signaling pathways. One of them, the Notch pathway, is important for the communication between neighboring cells, directing cell fate decisions^1^,^2^,^3^. In humans, defects in Notch signaling have been involved in a multitude of congenital diseases as well as in cancer formation^4^,^5^,^6^. The critical requirement of Notch signaling activity in cell-fate specification as well as maintenance of adult tissue homeostasis demands strict regulation of both activation and repression. Since the Notch pathway is highly conserved in eumetazoans, *Drosophila melanogaster* serves as a prime model allowing the *in vivo* manipulation of Notch signaling components along with the study of their immediate consequences^7^. Notch pathway activation commences with the binding of ligands (for example Delta in *Drosophila)* to the transmembrane receptor Notch. As consequence of ligand binding, Notch is cleaved and the Notch intracellular domain (NICD) is released. In the nucleus NICD assembles an activator complex on Notch target gene promoters together with Suppressor of Hairless [Su(H)] plus co-activators to transcriptionally activate Notch target genes^2^,^8^. Su(H) is central to Notch signal transduction; it is a transcription factor with the ability to activate or repress Notch target genes depending on interacting cofactors. In the absence of Notch activity these target genes are silenced by a repressor complex comprising Su(H) and Hairless (H), which additionally recruits the corepressors Groucho and dCtBP^9^,^10^,^11^,^12^,^13^.

In *Drosophila*, manifold developmental decisions require Notch pathway activity. For instance, in a process called *‘lateral inhibition’*, a unique precursor cell is singled out from a group of equipotent cells, thereby acquiring a specific cell fate. This can be nicely observed during the selection of sensory organ precursor cells that eventually form a mechano-sensory bristle^14^ or during the specification of vein width in the wing^15^. Notch signaling is turned on in the receiving cells that are forced into a secondary cell fate, non-neuronal in the case of the sensory organ and intervein fate in the case of the wing vein. Moreover, Notch activity is required for the formation of the wing margin: In heterozygous *Notch* mutant females the margin is defective, i.e. the wings are ‘*notched’*^16^,^17^. In the absence of the antagonist H, Notch activity is increased, resulting in weak Notch gain of function phenotypes. These notably affect the wing-i.e. causing vein gaps due to hyperactive lateral inhibition of vein fate-and the number and set up of mechano-sensory bristles-i.e. resulting in bristle loss and specific cell transformations^17^,^18^,^19^. As is the case with *Notch* and *∆*, mutations in *H* are haplo-insufficient, demonstrating the strong dose sensitivity of Notch signaling activity.

Given the finding of an in-frame internal ribosome entry site (IRES) in the *H* full length transcript^20^, contextual regulation of Notch signaling might also involve dynamically regulated translation of different H isoforms. Three H protein isoforms are derived from the *H* locus: two isoforms of about 150 kDa occur by conventional translation initiation (H^p150^), and one by internal ribosome entry. The IRES in the *Drosophila H* gene is peculiar because it is situated within the open reading frame. As a consequence, the N-terminally truncated smaller protein isoform H^p120^ is made in addition to the full length H^p150^ protein^20^ (Fig. 1, Supplementary Fig. S1). Overexpression of heat shock inducible transgenes encoding either just one or both isoforms revealed that both H^p150^ and H^p120^ isoforms are functional *in vivo*. Moreover, the results indicated translation of H^p120^ during mitosis, suggesting a requirement of H protein throughout the cell cycle^20^. In these experiments, however, endogenous H protein was still present in the analyses, limiting conclusions on possible specific functions of the different H isoforms in the native setting. We have recently established a *H* knock-out allele by genome engineering, that carries an attP landing site in place of the *H* locus^21^. Aiming at the functional analysis of the individual isoforms, this site was used to generate flies expressing just one H isoform or specific combinations of these isoforms. Our results indicate that each H isoform on its own is functional, with marked differences in activity: flies expressing just H^p150^ are nearly wild type, while IRES derived H^p120^ is less potent, and the resultant flies show many *H* mutant defects. Whereas many of the phenotypic variations may be explained by differences in protein levels, our data suggest a concomitant requirement of both isoforms during wing growth and vein width refinement. Furthermore, nuclear enrichment of H^p120^ in mitotic cells supports the notion of IRES mediated translation and the specific requirement of H protein during the entire cell cycle.

## Results

### Generation of new *Hairless* alleles by genome engineering

**Figure 1:**
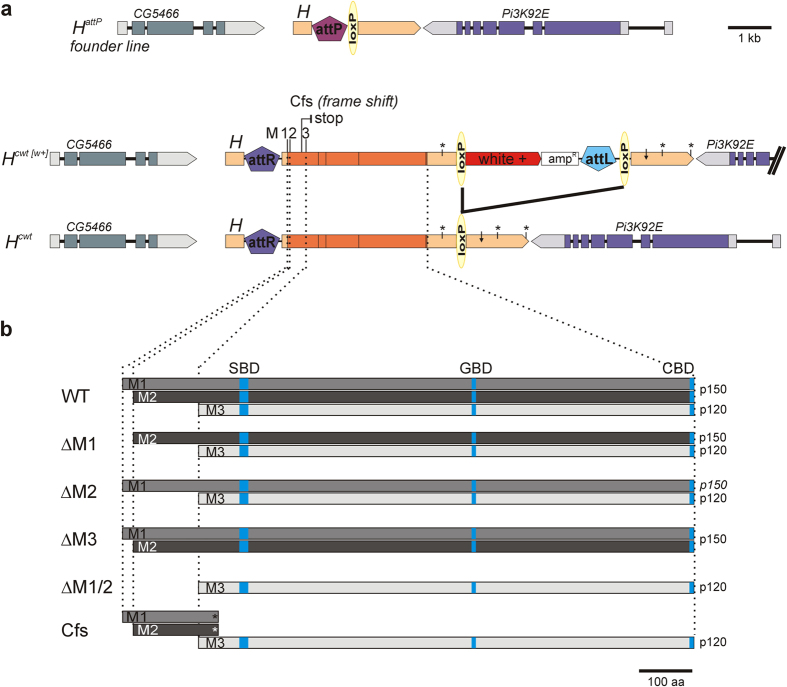
Structure of the *Hairless* locus and the IRES-constructs. (**a**) Sketch of the genomic organization of the *H*^*attP*^ founder line, of *H*^*cwt*[*w*+]^ containing the *white*^+^ marker gene in the 3′ UTR of the *H* gene, and of *H*^*cwt*^ generated by floxing out superfluous DNA. Coding sequences are in dark orange (with intron positions indicated by dashes); untranslated sequences are in light orange (with p(A)sites marked by asterisks, and miR-305 site^35^ by a small arrow). M1,2,3 indicate position of the first, second and third possible start codons Met_1_, Met_18_, Met_148_, respectively. Cfs denotes the frame shift mutation resulting in a premature stop codon as indicated. Sequences derived from the genome engineering process attR, attL, loxP and vector backbone (ampR) are not to scale. The neighbouring loci *CG5466* and *Pi3K92E* are sketched. Scale bar, 1 kb. (**b**) H protein variants derived from the wild type and mutant constructs are depicted; the Suppressor of Hairless binding domain (SBD), the Groucho binding domain (GBD) and the binding domain of the C-terminal binding protein (CBD) are indicated in light bluish colours. M1-M3 denote start codons as above; p150 and p120 the size of the corresponding protein in kDa. Scale bar, 100 amino acids (aa).

*Hairless (H)* encodes two major protein isoforms, of 150 kDa and 120 kDa apparent molecular weight (H^p150^ and H^p120^)^20^,^22^. The H^p150^ isoform arises by conventional translation initiation at Met_18_ (M2), corresponding to proteins PB and PC in flybase derived from transcripts RB and RC. In addition, initiation can also occur at the first methionine Met_1_ (M1) from a splice variant found in transcripts RA and RD (flybase) lacking intron 0 (see Supplementary Fig. S1). This slightly longer protein variant, however, is observed *in vivo* less frequently^20^, presumably because M1 conforms suboptimal to the translation initiation consensus^23^ (see Supplementary Fig. S1). All four *H* transcripts can give rise to H^p150^ by ribosome scanning starting at M2. In contrast, the H^p120^ isoform results from an internal ribosome entry site (IRES) positioned next to Met_148_ (M3) (see Supplementary Fig. S1)^20^. The IRES in the *Drosophila H* gene is peculiar, because it is situated within the open reading frame. As a consequence, the N-terminally truncated smaller protein isoform H^p120^ is made in addition to the full length H^p150^ protein (Fig. 1, see Supplementary Fig. S1)^20^.

In order to study the individual contribution of the various H protein isoforms we established specific *H* strains by genome engineering. To this end we used the *H*^*attP*^ founder line that carries an attP landing platform in place of the *H* coding region, whereas the *H* promoter and parts of the 5′ leader and 3′ trailer sequences are still present in the genome (Fig. 1a)^21^. A total of five different H constructs affecting individual start codons or a combination of them were introduced into the native *H* locus, giving rise to only specific H protein isoforms (Fig. 1b).

Constructs producing specific H protein isoforms were generated by mutation of the respective start codons (Fig. 1b): M1 (Met_1_), M2 (Met_18_) or M3 (Met_148_) were exchanged by codons for valine (GUG) to generate the mutant constructs ΔM1, ΔM2, ΔM1/2 and ΔM3. In addition, a frame shift mutation Cfs was introduced 35 codons upstream of M3, the start codon used for IRES mediated translation of H^p120^, as outlined before^20^. This frame shift results in a premature translation stop 45 amino acids downstream of M3 (Fig. 1b; see Supplementary Fig. S1)^20^. As a consequence, neither read-through nor re-initiation is possible in the Cfs construct^24^, and H^p120^ can arise exclusively through use of the IRES. The constructs were cloned into pGEattB^GMR^ that contains a *white*^+^ gene as selection marker^25^. This way, the fly lines *H*^*ΔM1*[*w*+]^, *H*^*ΔM2*[*w*+]^*, H*^*ΔM1/2*[*w*+]^*, H*^*ΔM3*[*w*+]^ and *H*^*Cfs*[*w*+]^ were generated (Fig. 1a). Since all constructs are based on the *H* cDNA, the *H*^*cwt*[*w*+]^ allele was used as control^21^.

In a second step, the *white*^+^ selection marker including accompanying vector DNA was eliminated by a Cre-loxP mediated recombination (Fig. 1a)^25^. The resultant strains *H*^*cwt*^*, H*^*ΔM1*^, *H*^*ΔM2*^*, H*^*ΔM1/2*^*, H*^*ΔM3*^ and *H*^*Cfs*^ regain the original structure of the locus, except for the introduced mutations, plus some additional sequences in the 5′ UTR and 3′ UTR^25^. Moreover, the constructs lack all *H* introns (Fig. 1a).

### Phenotypic analysis of the new *Hairless* alleles

#### Analysis of the unfloxed [w^+^] alleles

**Figure 2:**
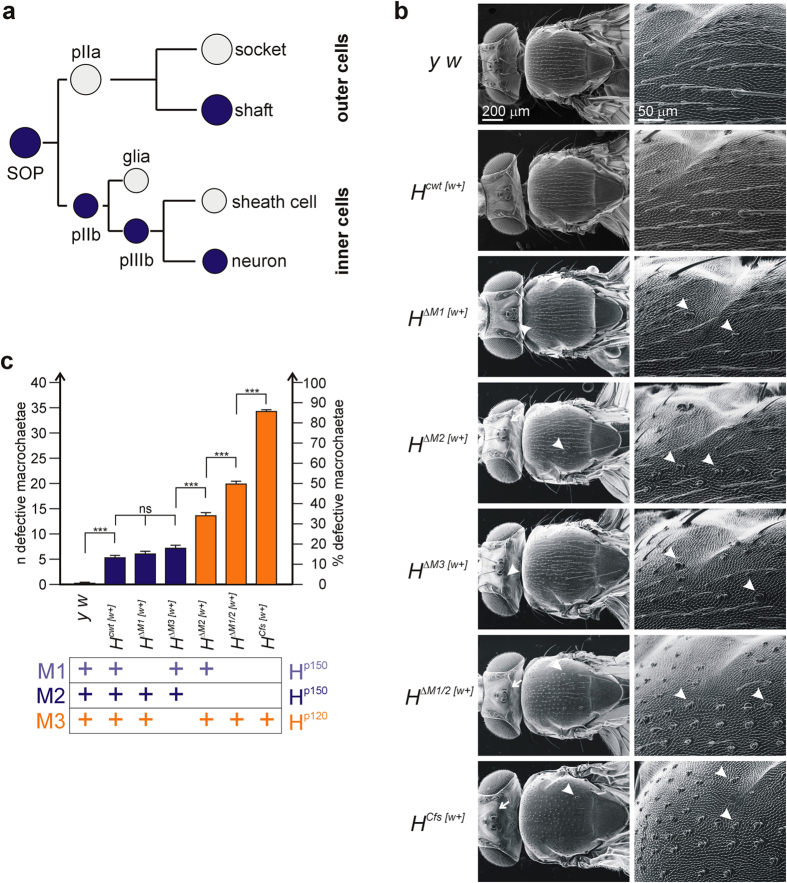
Adult bristle phenotypes of new *Hairless* alleles before floxing. (**a**) Scheme of bristle development starting with a sensory organ precursor (SOP) selected by lateral inhibition from a cluster of proneural cells. It generates two daughter cells (pIIa, b) determined for outer fates (socket and shaft cells), and inner fates (glia, sheath cell and neuron), respectively^26^. Cells labelled in blue require H activity for fate specification by protection from erroneous Notch activity^14^,^26^. (**b**) Scanning electron micrographs of adult homozygous flies of the indicated genotype. Control was *y*^*1*^ *w*^*67c23*^ (*y w*). Left panel shows an overview, right panel an enlargement of the upper anterior scutum. Arrows point to missing bristles on the head (notably postverticals are affected); arrowheads point to examples of double sockets resulting from a shaft to socket transformation. (**c**) Statistical evaluation of defective macrochaetae (missing or transformed to sockets) of each genotype by ANOVA using a two-tailed Tukey-Kramer approach for multiple comparisons (40 flies each were evaluated, and 20 in the control). Total number (n) as well as percentage of affected bristles is indicated. Note statistically significant bristle defects in *H*^*cwt*[*w*+]^ compared to *y*^*1*^ *w*^*67c23*^ control that match *H*^*ΔM1*[*w*+]^ and *H*^*ΔM3*[*w*+]^. Highly significant ***p < 0.001; not significant ns, p > 0.05. Increasingly stronger bristle defects are observed in *H*^*ΔM2*[*w*+]^*, H*^*ΔM1/2*[*w*+]^ and *H*^*Cfs*[*w*+]^ that all fail to produce H^p150^ protein. Fly lines unable to produce H^p150^ from M2 are depicted in orange. The scheme below depicts the H protein isoforms present in the different alleles.

All of the new *H* strains are homozygous viable and fertile. Whereas the heterozygous flies had a wild type appearance, the homozygotes displayed bristle defects typical for *H* loss of function^18^,^21^: Firstly, lack of H activity may result in a complete absence of the entire bristle organ as a consequence of the failure to protect the sensory organ precursor cell (SOP) from lateral inhibition by Notch (Fig. 2a,b). Secondly, shaft to socket transformations are frequently observed, typified by a double socket phenotype (Fig. 2a,b). These result from a failure to correctly distinguish between alternative bristle cell fates^26^.

To better characterize the lines, the number of affected macrochaetae was determined (Fig. 2c). A wild type fly has a total of 40 large bristles on head and thorax, which are typically affected in *H* mutants^18^. The control line *H*^*cwt*[*w*+]^ displays a mild *H* loss of function phenotype with about five macrochaetae affected on average (Fig. 2b,c), presumably as a result of the *white*^+^ gene within the *H* 3′ UTR. Both major transcripts RA and RC use a poly(A) site downstream of these integrated sequences, i.e. their processing might be hampered (Fig. 1a). Similar mild phenotypes were observed in *H*^*ΔM1*[*w*+]^ and *H*^*ΔM3*[*w*+]^ lines that still express the H^p150^ isoform from M2: Here, six or seven macrochaetae are affected on average (Fig. 2b,c). *H*^*ΔM2*[*w*+]^ flies have a significantly stronger phenotype, indicating that H^p150^ derived from M2 has the strongest impact on bristle development. The most extreme phenotypes were observed in *H*^*ΔM1/2*[*w*+]^ and *H*^*Cfs*[*w*+]^ fly lines that only express H^p120^: 20 to 37 macrochaetae are affected on average (Fig. 2b,c). Especially striking was the near complete shaft to socket transformation of most of the macro- and microchaetae in the *H*^*Cfs*[*w*+]^ flies (Fig. 2). Apparently, H^p120^ is less active than H^p150^ although it is sufficient for fly survival. The large difference between *H*^*ΔM1/2*[*w*+]^ and *H*^*Cfs*[*w*+]^ may be explained by a read-through in the former line, which is precluded in the latter, indicating that quantitative differences are pivotal to phenotypic strength.

The following phenotypic series from mild to strong bristle phenotypes was built: *H*^*cwt*[*w*+]^ <*H*^*ΔM1*[*w*+]^ *<H*^*ΔM3*[*w*+]^ *<H*^*ΔM2*[*w*+]^ <*H*^*ΔM1/2*[*w*+]^ <*H*^*Cfs*[*w*+]^. Wing venation was almost normal, except for small gaps in the fifth longitudinal wing vein in many *H*^*Cfs*[*w*+]^ flies.

#### Analysis of the floxed [w^−^] alleles

**Figure 3:**
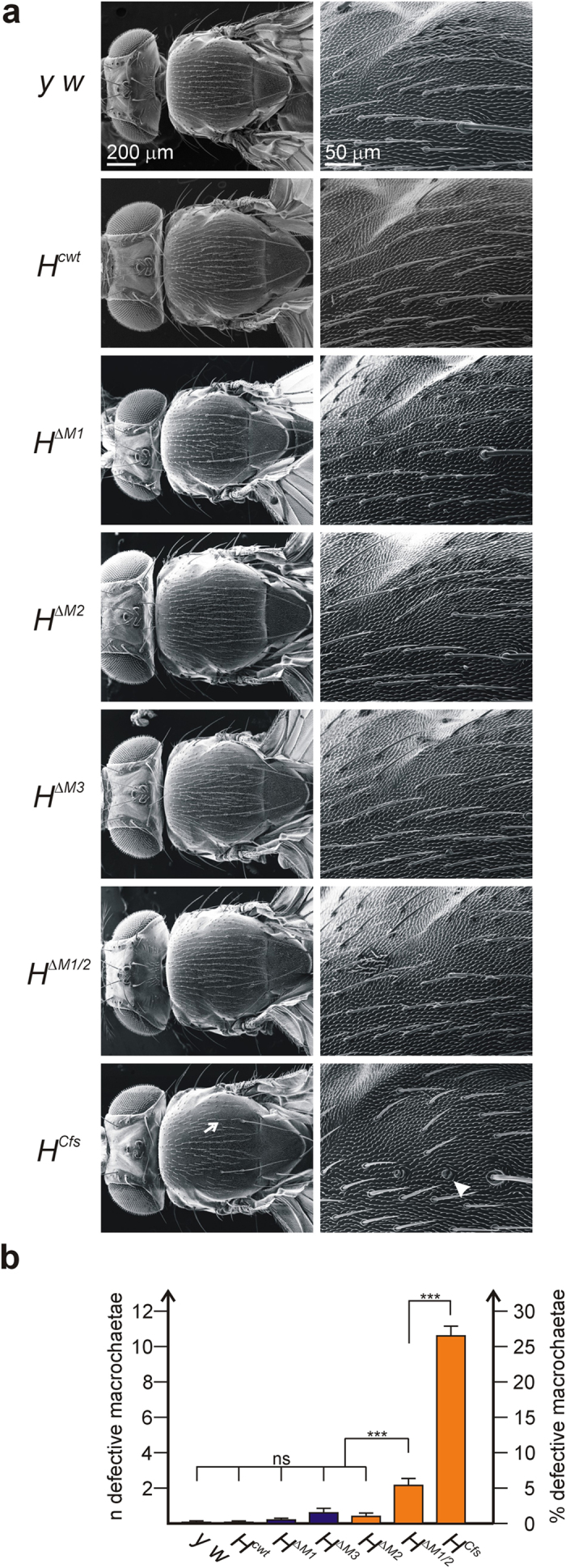
Adult bristle phenotypes of new *Hairless* alleles after floxing. (**a**) Scanning electron micrographs of adult homozygous flies of the indicated genotype. Control was *y*^*1*^ *w*^*67c23*^ (*y w*). Left panel shows an overview, right panel an enlargement of the upper anterior scutum. Arrow points to example of bristle loss; arrowheads to double sockets resulting from a shaft to socket transformation in *H*^*ΔM1/2*^ and *H*^*Cfs*^, respectively. (**b**) Statistical analysis of defective bristles of each genotype by ANOVA using a two-tailed Tukey-Kramer approach for multiple comparisons (n = 20 flies). Fly lines unable to produce H^p150^ from M2 are depicted in orange. There is no statistically significant difference between *H*^*cwt*^*, H*^*ΔM1*^, *H*^*ΔM3*^ nor *H*^*ΔM2*^ compared to *y*^*1*^ *w*^*67c23*^control. Significant bristle defects are only observed in *H*^*ΔM1/2*^ and even more in *H*^*Cfs*^ homozygotes. Highly significant ***p < 0.001; not significant ns, p > 0.05.

Because of its disruptive effects, we eliminated the *white*^+^ marker gene and vector DNA by the Cre-loxP mediated recombination and repeated our phenotypic analysis. After removal of the superfluous sequences, homozygous stocks were established that lacked remarkably less bristles compared to the unfloxed lines (Figs 2 and 3). No wing phenotypes were seen (see below). The lines *H*^*cwt*^, *H*^*ΔM1*^, *H*^*ΔM3*^ and *H*^*ΔM2*^ are within the range of the *y*^*1*^ *w*^*67c23*^ control flies used for the generation of the founder line (Fig. 3b). The most striking improvement was seen for the phenotype of the *H*^*Cfs*^ line: almost all bristle shafts formed normally. The phenotypic series, however, was unchanged: *H*^*cwt*^ < *H*^*ΔM1*^* < H*^*ΔM3*^* < H*^*ΔM2*^ < *H*^*ΔM1/2*^* < H*^*Cfs*^.

#### Analysis of hemizygotes

**Figure 4:**
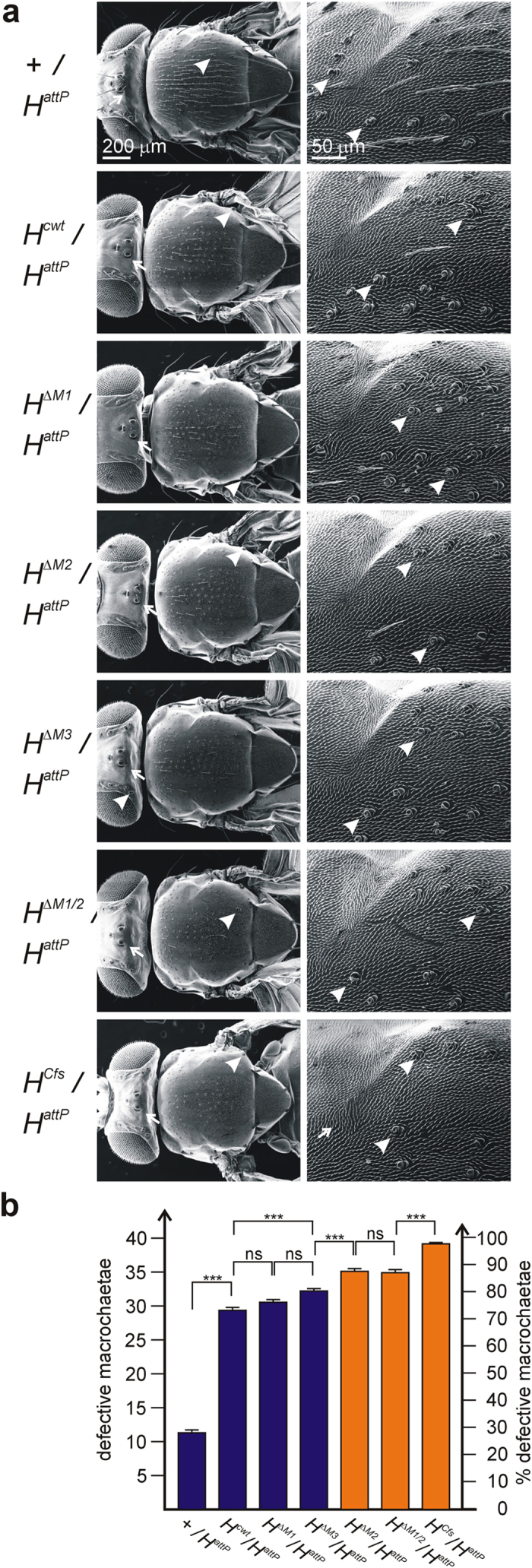
Adult bristle phenotypes of new *Hairless* alleles in hemizygosis. (**a**) Scanning electron micrographs of adult flies of the indicated genotype *in trans* over the null allele *H*^*attP*^. Control was heterozygous +/*H*^*attP*^ in the background of *y*^*1*^ *w*^*67c23*^. Left panel shows an overview, right panel an enlargement of the upper anterior scutum. Arrows point to examples of missing bristles; arrowheads to examples of double sockets resulting from a shaft to socket transformation. Note the absence or transformation of several macro- and microchaetae. (**b**) Statistical analysis of defective bristles of each genotype by ANOVA using a two-tailed Tukey-Kramer approach for multiple comparisons. Bars denote standard error with n = 20 flies. Fly lines unable to produce H^p150^ from M2 are depicted in orange. Note statistically significant bristle defects in *H*^*cwt*^*/H*^*attP*^ compared to *y*^*1*^ *w*^*67c23*^ control. The differences between the other genotypes are small, however, still significant between hemizygous *H*^*cwt*^ and *H*^*ΔM3*^, *H*^*ΔM3*^and *H*^*ΔM2*^ as well as *H*^*ΔM1/2*^and *H*^*Cfs*^. Highly significant ***p < 0.001; not significant ns, p > 0.05.

We next addressed the question, whether one copy of each of the individual H isoforms was sufficient for fly development. Fly viability in hemizygosis was addressed *in trans* over the *H* knock-out founder line *H*^*attP*^. As *H* is strictly dose dependent, the hemizygous condition corresponds to a highly sensitized background highlighting any residual *H* activity^21^. To this end we used the floxed alleles to exclude any negative effects of impeded mRNA processing. The lines were crossed to *H*^*attP*^ and phenotypes were recorded (Fig. 4).

All new *H* alleles were viable in hemizygosis. *H*^*Cfs*^/*H*^*attP*^ flies, however, hatched with lower frequency compared to their siblings, although the number of pupae was normal, indicating that the animals developed normally through larval stages but were at a disadvantage during metamorphosis (see Supplementary Fig. S2). All others matched the control (see Supplementary Fig. S2). As expected, the *H* mutant phenotype was much stronger in the hemizygotes than in the homozygotes (Fig. 4). Whereas the phenotypic series remained largely the same, we noted some interesting differences in the relative strength of the phenotypes. For example, there was no significant difference between *H*^*cwt*^, *H*^*ΔM1*^ and *H*^*ΔM3*^ in support of the notion that H^p150^ derived from M2 is the most important protein isoform for bristle formation. Likewise the similarity between *H*^*ΔM2*^ and *H*^*ΔM1/2*^ suggests M1 contributes very little to H^p150^ protein translation. The phenotype of *H*^*Cfs*^ was clearly stronger than that of *H*^*ΔM1/2*^ indicative of additional H activity maybe derived from read through instead of exclusive internal ribosome entry (Fig. 4b).

### The new *Hairless* alleles produce the expected specific Hairless isoforms

**Figure 5:**
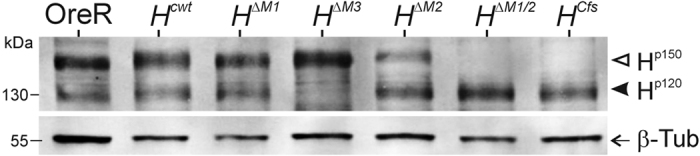
Hairless Protein expression in the new *Hairless* alleles. Protein extracts derived from homozygous embryos as indicated were probed for H protein expression; Oregon R served as wild type control. The isoforms H^p150^ (open arrowhead) and H^p120^ (closed arrowhead) are indicated. Beta-Tubulin (ß-Tub) was used as loading control (arrow) on the same blot that was cut apart. The figure was cropped to show the relevant bands; the full-length blot is presented in Supplemental Fig. 3. Approximate size is given in kilo Dalton (kDa).

The new *H* alleles were confirmed to produce just the specific H protein isoforms (Fig. 5): As predicted from our earlier work, H^p150^ is the major isoform derived from M2. It is present in *H*^*cwt*^, *H*^*ΔM1*^ and *H*^*ΔM3*^. Also M1 is used, but at lower levels as revealed by the *H*^*ΔM2*^ protein extract. Expression of H^p120^ from M3 appears somewhat lower than that of H^p150^; it is detected in all strains but *H*^*ΔM3*^. As expected, the *H*^*ΔM1/2*^ line expressed only the 120 kDa protein. One may argue that this is through ribosomal scanning to the first suitable AUG codon in the absence of M1 and M2. Nevertheless, H^p120^ is also detected exclusively in the *H*^*Cfs*^ protein extracts at a similar level. Since read-through is prevented by the frame shift mutation^20^,^24^, the Cfs mRNA can only be translated by use of internal ribosome entry: M1 and M2 may also be used for translation initiation, but translation stops early after 190 codons as a consequence of the frame shift mutation introduced in this construct (see Supplementary Fig. S1)^20^. Interestingly, expression of H^p120^ in *H*^*ΔM1/2*^ and *H*^*Cfs*^ is not very different suggesting that IRES mediated translation is the main process of H^p120^ generation (Fig. 5).

### Requirement of the two Hairless protein isoforms for growth regulation of the wing

**Figure 6:**
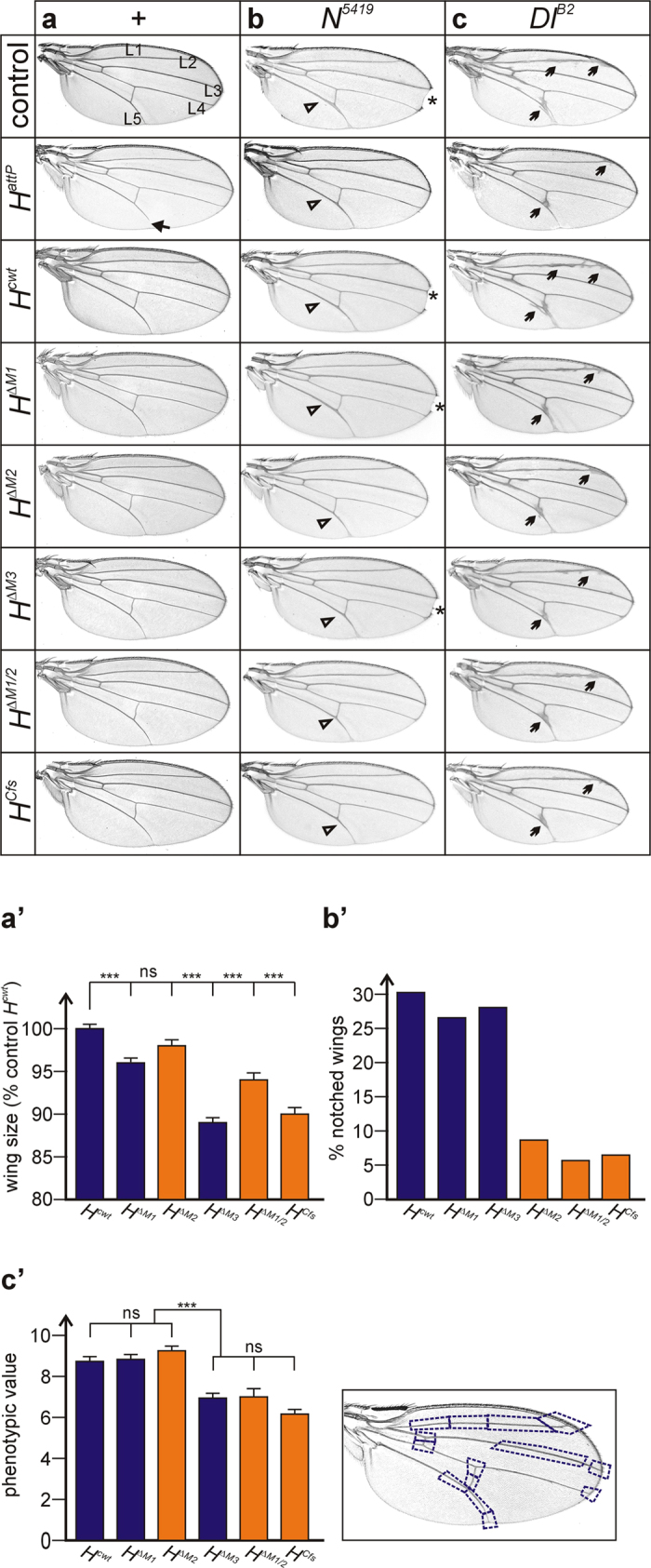
Genetic interactions with *N*^*5419*^ and *Dl*^*B2*^ mutants. (**a**) Wings of female flies; the longitudinal veins L1-L5 are indicated in the control *y*^*1*^ *w*^*67c23*^. Flies are homozygous except for the null allele *H*^*attP*^: arrow points to shortened L5 longitudinal vein in the wing of *H*^*attP*^. Note intact veins in the new *H* alleles. (**b**) Wings of female flies heterozygous for the given *H* allele (indicated at left) and *Df(1)N*^*5419*^. Control is heterozygous *y*^*1*^ *w*^*67c23*^/*Df(1)N*^*5419*^. Open arrowheads point to examples of thickened veins; asterisks denote wing notches. (**c**) Wings of female flies trans-heterozygous for the given *H* allele and *Dl*^*B2*^. Control is heterozygous +/*Dl*^*B2*^. Arrows point to examples of thickened veins. (**a’**) Wing size was determined from homozygous female flies of the given genotype, and is presented as percentage of the control; *H*^*cwt*^ was taken as 100%. Genotypes unable to produce the major H^p150^ isoform are labelled in orange. Bars denote standard error with n = 40. (**b’**) Percentage of notched wings displayed by *N*^*5419*^ heterozygotes while bearing the *H* allele indicated (genotype *N*^*5419*^/+; H^*^/+) (n ≥ 120). Genotypes unable to produce the major H^p150^ isoform from M2 are labelled in orange (*H*^*ΔM2*^, *H*^*ΔM1/2*^, *H*^*Cfs*^); they show significantly less notched wings than the other group (*H*^*cwt*^, *H*^*ΔM1*^, *H*^*ΔM3*^). (**c’**) Right panel: Veins were subdivided into 14 sectors (boxed) as shown, and thickening within each sector was monitored. If present, a value of 1 was given, and a value of 0 in the absence of thickening. Left panel: At least 15 wings of each genotype as indicated were compared; standard error is denoted by bars. Genotypes unable to produce the major H^p150^ isoform are labelled in orange. Note that *H*^*ΔM2*^ behaves similar to *H*^*cwt*^ and *H*^*ΔM1*^, whereas *H*^*ΔM3*^ is more similar to *H*^*ΔM1/2*^ and *H*^*Cfs*^. The two groups are significantly different with regard to the amelioration of the *Delta* wing venation phenotype.

H acts as the major antagonist of Notch in *Drosophila*. Hence, a loss of H activity equals a gain of Notch activity^11^ which is apparent in the wing. For example, the L5 longitudinal vein is typically shortened in *H* heterozygotes^17^,^21^,^27^,^28^ which, however, is not seen in any of the mutants (Fig. 6a). In addition, wings from heterozygous *H* mutant animals are about 5% larger than controls^29^. The new *H* alleles were more similar to the control *H*^*cwt*^, except for *H*^*ΔM3*^ and *H*^*Cfs*^ which displayed considerably smaller wings reaching only about 90% of the control (Fig. 6a’). This finding was unexpected as it suggests that wing growth relies on IRES derived H^p120^ in the case of *H*^*ΔM3*^ and likewise on H^p150^ in the case of *H*^*Cfs*^. Apparently, both H protein isoforms are required concomitantly for growth regulation of the adult wing.

### Genetic interactions of *Hairless* with *Notch* and *Delta* mutants

Similar to *H, Notch (N)* and *Delta (Dl)* mutants are haplo-insufficient displaying dominant wing phenotypes; *N* mutant wings are notched and longitudinal veins L3 and L5 are thickened, *Dl* mutant wings display thickened veins ending in deltas at the wing margin^15^,^16^ (Fig. 6b,c). It is well known that *in trans* over *H*, heterozygous *N* as well as *Dl* mutant phenotypes are abrogated^17^,^21^,^28^. We wanted to know, whether both H isoforms likewise contribute to the H antagonistic activity, or whether alleles expressing exclusively one or the other isoform behaved differently in the combination with *N*^*5419*^ or *Dl*^*B2*^ loss of function mutants. A rescue of *N* or *Dl* wing phenotypes was expected for those alleles that lacked H activity, whereas the ones with a strong residual H activity should be more similar to the control.

Indeed, rescue of the *N* wing phenotype correlated well with the bristle phenotypes displayed by homo- and hemizygous *H* alleles; the best rescue of notching was seen with those alleles that displayed the strongest *H* phenotypes and thus had the least bristle numbers, for example, *H*^*ΔM1/2*^ or *H*^*Cfs*^ (Figs 2b and 6b,b’). The alleles fell into two groups, one behaving similar to the control (*H*^*cwt*^, *H*^*ΔM1*^, *H*^*ΔM3*^) – the group that can produce H^p150^ protein from M2 – and the other rescuing well, i.e. the ones with less H activity (*H*^*ΔM2*^, *H*^*ΔM1/2*^, *H*^*Cfs*^) (Fig. 6b,b’).

In contrast, the alleles behaved differently in rescuing the *Dl* phenotype. For example, *H*^*ΔM3*^ was similar to *H*^*ΔM1/2*^ or *H*^*Cfs*^, whereas *H*^*ΔM2*^ matched *H*^*cwt*^ or *H*^*ΔM1*^ (Fig. 6c,c’). Again, two phenotypic groups were obtained, one with little influence on *Dl* wing knotting, resembling the control (*H*^*cwt*^, *H*^*ΔM1*^, *H*^*ΔM2*^); the other one with a stronger rescue, reflecting a loss of H activity (*H*^*ΔM3*^, *H*^*ΔM1/2*^, *H*^*Cfs*^). The two groups differ in their ability to produce both H protein isoforms simultaneously: only the former can do so, whereas the latter can produce just either the long H^p150^ or the short H^p120^ isoform. This is another indication of a process where both H protein isoforms are needed at the same time, and may relate to the regulation of Notch signaling by cis-inhibition^30^.

### The H^p120^ isoform is enriched in the nuclei of mitotic cells

**Figure 7:**
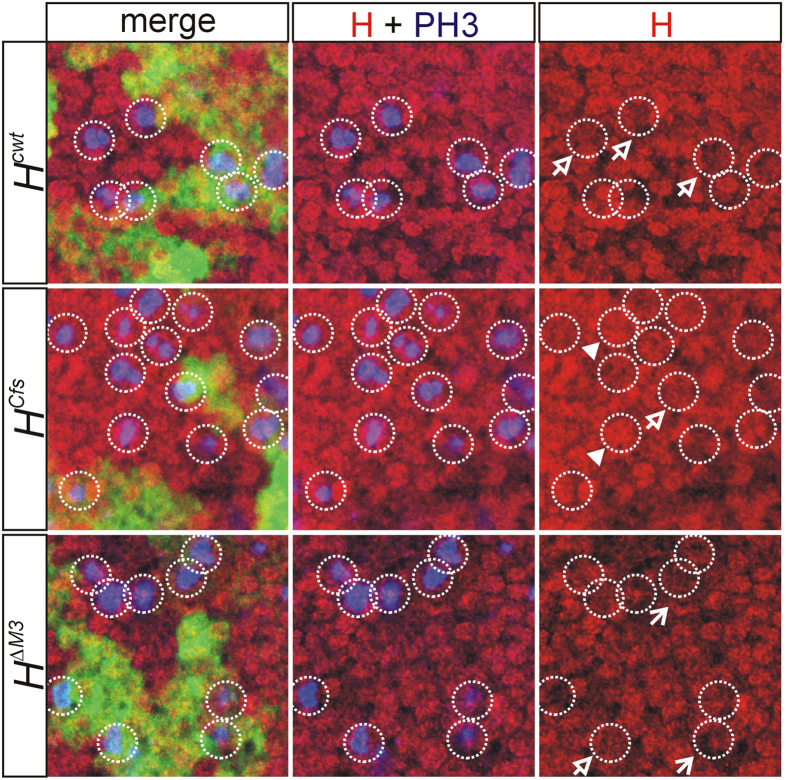
Nuclear enrichment of Hairless protein in mitotic nuclei. Clonal analysis in wing discs was performed using the given *H* allele: cells homozygous for the wild type chromosome express GFP strongly (labelled green in the merge panel), heterozygous cells weakly, and cells homozygous for the given *H* allele lack GFP completely. The discs were stained for H protein (H, red) and phosphorylated Histon H3 (PH3, blue) as mitotic marker. Mitotic cells are encircled. Open arrows point to examples of wild type nuclear H staining. Closed arrowheads point to examples of H enrichment in mitotic nuclei; small arrows to examples of reduced H staining.

IRES dependent translation occurs as a response to cellular stress which induces a general inhibition of protein synthesis. Protein synthesis is also stopped with the onset of mitosis, however, still allowing the translation from internal ribosomal entry^31^. We have shown before that the H^p120^ isoform derived from IRES mediated translation is enriched in mitotic cells when overexpressed in *Drosophila* tissues, in contrast to the H^p150^ isoform^20^. From this we concluded that H protein might be required throughout the cell cycle to regulate Notch signaling activity. As these experiments were based on a tissue specific overexpression, they may not reflect the biological relevance of H^p120^ and IRES usage. If an important function of the IRES is indeed production of H^p120^ during mitosis, we expect an enrichment of H^p120^ versus H^p150^ in mitotic cells. In this case H protein should be detected in the nuclei of mitotic cells in the *H*^*Cfs*^ strain which can produce H^p120^ protein only by IRES usage, but less so in the *H*^*ΔM3*^strain containing only H^p150^ protein which is made by conventional translation initiation during the interphase of the cell cycle. To address this hypothesis, a clonal analysis was performed in wing imaginal discs using either *H*^*cwt*^, *H*^*ΔM3*^ or *H*^*Cfs*^ alleles. The goal was to generate cell clones homozygous for the given allele, which produced either H^p120^ only (*H*^*Cfs*^ clones), or H^p150^ only (*H*^*ΔM3*^ clones), or both (*H*^*cwt*^ clones). Neighboring cells were always wild type being heterozygous for a wild type allele. The tissues were stained for H protein and for Histone H3 phosphorylated at Ser28 as a marker for mitotic cells as well as for GFP to outline clone borders. As shown in Fig. 7, control *H*^*cwt*^ cells contained H protein within mitotic nuclei, however to a lesser degree than cells homozygous for *H*^*Cfs*^, suggesting enrichment of IRES-derived H^p120^ in the latter. In contrast, little H protein accumulation was observed in the mitotic nuclei of cells homozygous for *H*^*ΔM3*^, in agreement with the idea that mitosis inhibits cap-dependent translation^31^,^32^.

**Figure 8:**
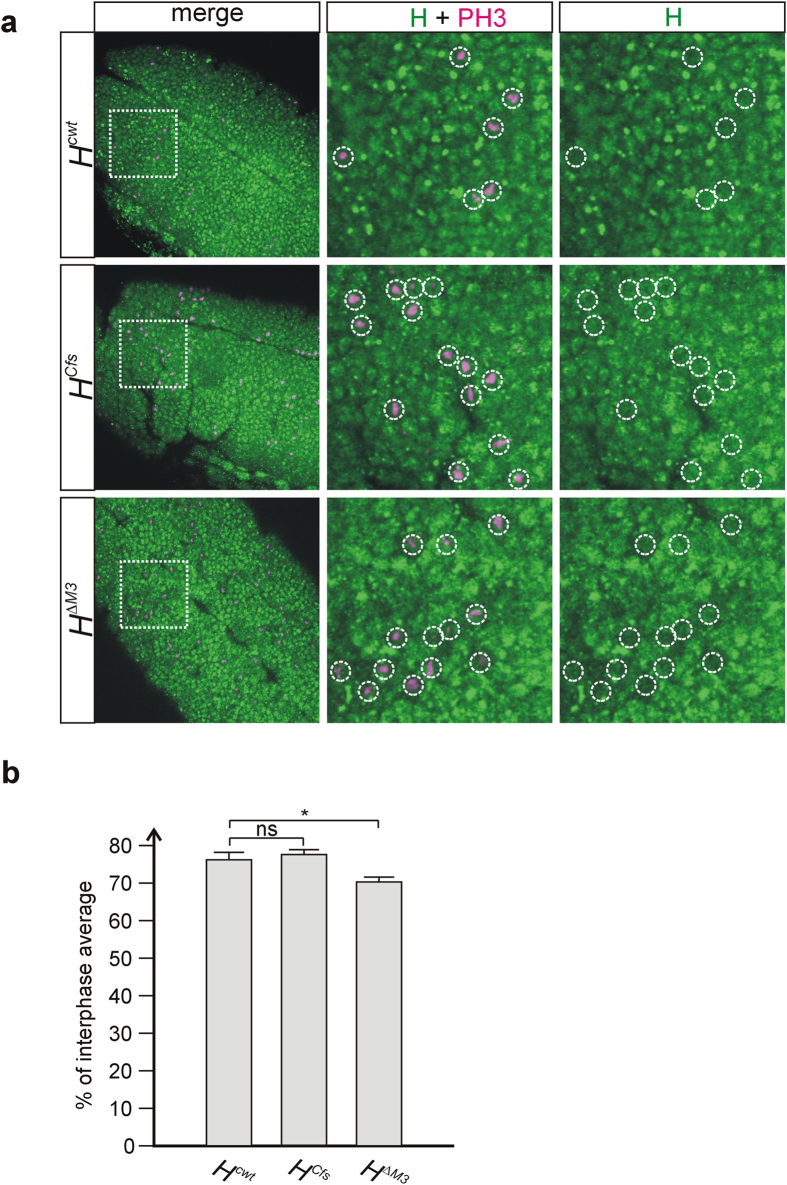
Nuclear enrichment of Hairless protein in mitotic nuclei of embryos. (**a**) Embryos homozygous for the given *H* allele were stained for H protein (H, green) and phosphorylated Histon H3 (PH3, magenta) as mitotic marker. A ventral surface view is shown of similarly staged embryos (stage 9–11). Boxed area corresponds to the enlargements shown in the central and the right panels; mitotic cells are encircled. The central panel shows the merge, the right panel just H protein staining. Note relative absence of H protein in mitotic nuclei primarily in *H*^*ΔM3*^. (**b**) H protein values were determined as % signal intensity by comparing the signal from mitotic nuclei with those from non-mitotic nuclei. (*H*^*cwt*^ n = 65, *H*^*Cfs*^ n = 74, *H*^*ΔM3*^ n = 67).

In an attempt to quantify the differences, we turned to embryos that in early developmental stages (stages 5–10) display isolated groups of synchronously dividing cells, the so-called ‘mitotic domains’, at discrete locations^33^, thereby allowing a more direct comparison of the different specimens. Embryos homozygous for either of the three *H* alleles, *H*^*cwt*^, *H*^*ΔM3*^ or *H*^*Cfs*^ were stained for both H protein and phospho-histone H3 (Fig. 8a). H protein of the mitotic cells and directly neighboring non-mitotic cells was quantified: a statistically significant enrichment of nuclear H protein accumulation in mitotic nuclei of *H*^*Cfs*^ homozygous cells was detected compared to *H*^*ΔM3*^(Fig. 8b). These results strongly support the notion that H^p120^ is produced by internal ribosome entry throughout the cell cycle including mitosis.

## Discussion

The *Hairless* locus encodes two H protein isoforms, one with an apparent molecular weight of 150 kDa, and the other with 120 kDa. H^p150^ is the major isoform: it comprises 1058/1076 amino acids and originates from conventional translation initiation (see Supplementary Fig. S1)^20^,^28^. The N-terminally truncated H^p120^ is derived from internal ribosome entry at the third AUG codon in frame at position 148^20^. In addition to the canonical translation initiation site^23^ at the second methionine (Met_18_), the *H* gene contains a *bona fide* IRES within the coding region around codon 135 (see Supplementary Fig. S1)^20^,^33^,^34^. This IRES is active in heterologous assays, and directs translation from the third methionine (Met_148_)^20^. Our earlier work indicated functional activity of both H protein isoforms and a specific production of H^p120^ in mitotic cells of imaginal tissues^20^. This work was extended employing the recently developed method of genome engineering^21^,^25^ to directly manipulate the *H* locus to produce specific H protein isoforms.

Several conclusions can be drawn from our results, regarding the requirement of the *H* 3′ UTR, the equivalence of the two H protein isoforms and their combined activity during wing development. Finally we provide evidence for endogenous H^p120^ translation during mitosis *in vivo*.

Firstly, we note that disruption of the *H* 3′ UTR by a *white*^+^ marker gene strongly affects *H* function (Fig. 2). *H* transcripts differ in length of their 3′ UTR by usage of different poly(A)sites^27^,^28^. The insertion of *white*^+^ and vector sequences interrupts 3′ UTR transcription enforcing termination at the earlier poly(A)sites (Fig. 1). Presumably, the early termination is less efficient which may explain reduced *H* activity in the strains containing the *white*^+^ marker gene. We note that the respective H^*[*w*+]^ fly lines are homozygous viable despite some bristle defects, indicating that the long 3′ UTR is not an essential requirement for *H* activity. The 3′ UTR of *H* contains a binding site for miR-305 suggested to negatively regulate *H* activity^35^. The miR-305 site, however, is downstream of the intervening sequences in the H^*[*w*+]^ alleles thus unable to exert its regulatory activity in these flies (Fig. 1).

Secondly, we find that all of the new *H* alleles are homozygous viable, and after floxing (i.e. without the long intervening sequences in the 3′ UTR) phenotypically indistinguishable from wild type with the exception of *H*^*ΔM1/2*^ and *H*^*Cfs*^ that show a weak *H* loss of function phenotype (Fig. 3). We conclude that either H^p120^ is not fully equivalent to H^p150^, or that H^p120^ does not reach the levels required for normal bristle development. Moreover, we found a strong requirement for H^p150^ when assessing the lines in hemizygosis (Fig. 4), i.e. with only one copy of the respective allele present. In summary, these results suggest that the H protein isoforms are largely interchangeable; the main difference between the alleles with respect to bristle phenotypes appears to be the amount of H protein, i.e. that the differences are rather quantitative than qualitative.

Thirdly, we have indications of a combined requirement of the two H protein isoforms during wing development. Notably wing growth is strongly dependent on the presence of both isoforms, since both *H*^*ΔM3*^and *H*^*Cfs*^ fly lines reach just 90% of the wing size of the control, and also *H*^*ΔM1/2*^has clearly smaller wings (Fig. 6a’). These lines can produce just one isoform – be it H^p120^ or H^p150^ – whereas all the other lines produce both. In addition to growth regulation, the process of cis-inhibition of Notch signaling activity may also depend on the combined activity of both H isoforms. This was concluded from the genetic interactions with the *Dl*^*B2*^ mutant (Fig. 6c,c’) that were strongest with those lines producing just one isoform, *H*^*ΔM3*^, *H*^*ΔM1/2*^ and *H*^*Cfs*^. In this assay the width of the wing veins was evaluated which is determined by Notch signaling activity. Thereby, Delta-Notch restricts the initial pro-vein territory to the vein proper^36^. Wing width is regulated in a complex molecular circuitry^37^, presumably involving cis-inhibition. Whenever the Delta ligand and the Notch receptor are expressed within the same cell, signaling activity is adjusted by mutual inhibition or titration, presumably enforcing the directionality of the Delta-Notch signaling process^30^. We propose that regulation of cis-inhibition requires both H protein isoforms based on the genetic interaction studies. Analyses of further developmental processes, for example eye development, may help to unravel the underlying mechanisms^30^.

Finally, our data support the notion that H^p120^ protein is indeed translated during mitosis since we find it enriched in mitotic nuclei in embryos and imaginal tissues alike (Figs 7 and 8). We have shown before^20^ that overexpressed H^p120^ specifically accumulates in mitotic nuclei unlike overexpressed H^p150^. Our data now suggest the same for endogenous H^p120^ protein. During interphase presumably both H protein isoforms are produced: H^p150^ by cap-dependent translation and H^p120^ by internal ribosome entry (in addition to potential read through). As we have no indication of H being a particularly unstable protein^20^,^38^, we expect both protein isoforms well into mitosis. Hence, enrichment of H^p120^ is a strong indicator of endogenous *H* mRNA translation during this phase of the cell cycle. With the onset of mitosis, transcription is aborted in *Drosophila*^39^ and cap-mediated translation is strongly inhibited^31^,^33^,^40^. Ornithin decarboxylase as well as PITSLRE kinase, two proteins required during mitosis, however, are produced in G2/M phase by cap-independent internal ribosome entry^33^,^41^. Accordingly, it has been generalized that cellular mRNAs may harbor an IRES to allow protein production at periods opposing translation, i.e. during mitosis, viral infection, stress or apoptosis^42^,^43^, although this idea has been challenged more recently^44^,^45^. Based on this reasoning we propose a general requirement of H protein throughout the cell cycle which is secured by the IRES in the *H* gene.

*H* encodes the major antagonist of Notch in *Drosophila*^11^. As a result, heterozygous *H* mutant flies display typical Notch gain of function phenotypes and are, for example, generally larger than wild type. In a hyperactive state, Notch can induce hyperplasia of imaginal tissues^46^. In this context, Notch activity stimulates growth by a direct transcriptional regulation of metabolic genes^29^. Interconnection of Notch and Insulin receptor (InR) pathways is hence to be expected, although only few and indirect data have been collected in *Drosophila*^35^,^47^,^48^,^49^. Interestingly, InR is translated by an IRES-mediated, 4E-BP-insensitive mechanism *in vivo* in *Drosophila*, thereby coupling transcription/translation that amplifies InR activation^50^. Cap-independent translation of H^p120^ protein is expected to occur also under nutrient deprived conditions, resulting in a repression of Notch activity in this state thereby helping to maintain cellular homeostasis as well.

## Methods

### Plasmid construction and genome engineering

Generation of knock out allele *H*^*attP*^ and respective constructs for reintegration into the *H* locus was according to^25^ and analogous to what was described in detail previously^21^. Briefly, construct H-cwt (containing *H* cDNA from position 118 to 4320 according to^28^ subcloned in pBT vector) was used for QuikChange site-directed mutagenesis (Agilent, California, USA) to produce constructs H-ΔM1, H-ΔM2, H-ΔM3 and H-ΔM1/2 using primers M1V, M2V and M3V, respectively (see Supplementary Fig. S1). H-Cfs was generated by deletion of 7 bases at the *Afl* II restriction site between M2 and M3 resulting in a frame shift from b to c^20^. All constructs were subsequently cloned as *Kpn* I fragments into pGE-attB^GMR^ as described^21^ and sequence verified. Transgenic flies were established by microinjection as outlined before^51^.

### Immunohistochemistry and imaging

Embryos were dechorionated and fixed for 20 minutes in 10% formaldehyde overlayed with *n*-heptane at room temperature. Devitellinisation was achieved by adding chilled methanol and shaking vigorously. Embryos were washed twice with methanol before rehydrating in a step-wise manner with PBS and blocked with 4% normal goat serum. Imaginal discs from third instar larvae were fixed in 4% formaldehyde for 20 minutes before blocking.

Immunostaining of embryos was performed over night with anti-H B (directed against C-terminal fragment of H) and of wing discs with anti-H A (directed against central H fragment)^22^, with anti-Phospho-Histone H3 (Ser28) (#9713, Cell Signaling Technology, Massachusetts, USA), and anti-GFP (sc-8334, Santa Cruz Biotechnology, Inc., Texas, USA). Secondary antibodies fused to FITC, Cy3 and Cy5 respectively were used (Jackson ImmunoResearch, Pennsylvania, USA). Embryos and imaginal tissue were mounted in VectaShield (Vector Laboratories, California, USA) before being imaged on a Bio-Rad MRC1024 system running LaserSharp 2000 imaging software (Bio-Rad Laboratories, California, USA) coupled to a Zeiss Axioskop (Carl Zeiss AG, Oberkochen, Germany). Figures were assembled using Corel Draw and Photo Paint software.

### Statistical evaluation

Bristles were analyzed by scoring each of the 40 macrochaetae on head and thorax as either ‘normal’ or ‘absent/defective’^18^. For each genotype one representative independent fly line was chosen from which either 20 or 40 adult flies in equal proportions were being assessed. Size of 40 wings (without alula or hinge)^29^,^46^ from each genotype was measured in pixel using Image J. A total of five similarly staged embryos of each genotype were used for mitotic cell counts (n = 65–74): signal intensity of a given number of mitotic nuclei from one embryo was determined in relation to the average signal of the same number of non-mitotic nuclei from the same embryo. Results were evaluated for statistical significance by ANOVA using a two-tailed Tukey-Kramer approach for multiple comparisons (highly significant ***p < 0.001; very significant **p < 0.01; significant *p < 0.05; not significant ns, p > 0.05). Documentation of phenotypes was performed on adult female flies using a table-top scanning electron microscope (NeoScope JCM-5000; Nikon, Tokyo, Japan).

### Mosaicism and genetic interactions

Cell clones were generated by Flp/FRT recombination^52^. All fly lines were recombined with P{neoFRT}82B (BL2050) and subsequently crossed with *y w* flp^1.22^; P{neoFRT}82B P{Ubi-GFP^S65T^nls}3R/TM6B as outlined in^53^. Flipase was induced by a 30 minutes heat shock at 39 °C in first to second instar larvae. Imaginal discs were prepared from wandering third instar larvae.

For genetic interaction analyses homozygous transgenic flies were crossed with Df(1)N-5419/FM7c^17^ or *Dl*^*B2*^/TM6C^54^ to determine genetic influence of H isoforms on *Notch* and *Delta* phenotypes, respectively. Offspring was selected for absence of balancer chromosomes and scored for notched wing phenotype or ectopic veinlets. Wings were dehydrated in ethanol and mounted in Euparal (Roth GmbH, Karlsruhe, Germany). They were analyzed on a Zeiss Axiophot, coupled to either a Pixera PVC-100C or Pro300D camera (Pixera Corporation, California, USA). Images were recorded using Pixera Viewfinder or iWorks 2.0 software, respectively. Figures were assembled using Corel Draw and Photo Paint software.

### Western blotting

Embryonic extracts were prepared by homogenizing 3 mg of embryos in 100 μl RIPA buffer (150 mM NaCl, 50 mM Tris-HCl pH7.5, 1% Triton-X, Pierce^TM^ Protease Inhibitor Mini Tablet [Thermo Fisher Scientific, Massachusetts, USA]) on ice. From our unpublished data we know that the H^p120^ isoform is only expressed zygotically, we therefore aged embryos for at least 5 hours after egg deposition before protein extraction. PAGE was performed with a step gel consisting of 12% and 7.5% polyacrylamide. Blots were stained with anti-H A^22^ and anti-β-Tubulin (E7, developed by M. Klymkowsky, obtained from the DSHB, Iowa, USA) as loading control.

## Additional Information

**How to cite this article**: Smylla, T. K. *et al. In vivo* analysis of internal ribosome entry at the *Hairless* locus by genome engineering in *Drosophila. Sci. Rep.* **6**, 34881; doi: 10.1038/srep34881 (2016).

## Supplementary Material

Supplementary Information
