# Supplementary material for: In vivo analysis of internal ribosome entry at the Hairless locus by genome engineering in Drosophila
Source: Sci Rep. 2016 Oct 7;6:34881. doi: 10.1038/srep34881 (PMC5054391; doi:10.1038/srep34881)
Supplement: Supplementary Information [file srep34881-s1.pdf]

Supplementary Information to

In vivo analysis of internal ribosome entry at the *Hairless* locus by genome engineering

Thomas K. Smylla, Anette Preiss, Dieter Maier*

Universität Hohenheim, Institut für Genetik (240), Garbenstr. 30, 70599 Stuttgart, GERMANY

E-mail: dieter.maier@uni-hohenheim.de

Supplementary Figure S1: The start sites in *Hairless*

Supplementary Figure S2: Survival rate of hemizygotes

Supplementary Figure S3: Raw data: Westernblot from Figure 5

Supplementary Figure S1 — **The start sites in *Hairless***

```
KpnI
ggtaccgtgccccaaaacatgaaaatggtgctagacgctcctaacgccgtgccgagctgg
catattttgcttagcgacgccgtcggctccgtttttcccggccgcgaactttgcgcgagg

                          M1V AAGTG                Intron 0
aatttacaacaaaaacaatgaattaaATGgtatttaaattgtccatataatgcggtttgg
                          M1

              Intron 0                                GCCCTG
aaatgcgaaattgtgcctaacttgcccaatgcaaacgaatgtttctctttgaagGCCCTG
                                                       A  L        3

CTTAATGACG   M1V                   M2V   CAACAGTGACCGATGAGCAT
CTTAATGACGTCACAAGCGTAGCAGAGTGCAACAGACAGACAACAATGACCGATGAGCAT
L  N  D  V  T  S  V  A  E  C  N  R  Q  T  T  M18 T  D  E  H      23

AAAA  M2V
AAAAGTAACATTAACAGTAACAGCAGTCACTCCAGCAACAACAACAACAACGGCAGCAGC
K  S  N  I  N  S  N  S  S  H  S  S  N  N  N  N  N  G  S  S       43

AGCAATAACGACAACAACAGCAACGACGACGCAGCAAGTAGCAGCAACAGCAAAAACAAC
S  N  N  D  N  N  S  N  D  D  A  A  S  S  S  N  S  K  N  N       63

AACACCAGCAACGAGAGCAGCCACAGCAACAACAATACTAGTAGCATAATTGCAGAGGCG
N  T  S  N  E  S  S  H  S  N  N  N  T  S  S  I  I  A  E  A       83

GCCGCCAAGTTTCTACTGAAAAATGGCCTAAACGGCAGTAGCAGCACCAGCTACCCCCCT
A  A  K  F  L  L  K  N  G  L  N  G  S  S  S  T  S  Y  P  P      103

                        AflII
CTGCCACCGCCTCTGCCCGCCAACTTAAGCAGGACGACCACGCCCACGACAACGACAACG
L  P  P  P  L  P  A  N  L  S  R  T  T  T  P  T  T  T  T  T      123
                     K           G  R  P  R  P  R  Q  R  Q  R

CCCTCATCCTCCAGCTCCACCGCCTCAAATGGCTTTTTGCCGCATGCCAAGACGCCCAAA
P  S  S  S  S  S  T  A  S  N  G  F  L  P  H  A  K  T  P  K      143
 P  H  P  P  A  P  P  P  Q  M  A  F  C  R  M  P  R  R  P  K

M3V  TAGCATTGTGGCTGCGTCCG  M3V
AGTAGTAGCATTATGGCTGCGTCCGCCGCAGTGGCAGCCAGCGTCGTTGGAGCTACTGCG
S  S  S  I  M148 A  A  S  A  A  V  A  A  S  V  V  G  A  T  A    163
 V  V  A  L  W  L  R  P  P  Q  W  Q  P  A  S  L  E  L  L  R

TCCAAGCCCACCATCGATGTCCTGGGGGGCGTCCTGGACTACAGTTCCTTGGGCGGAGCT
S  K  P  T  I  D  V  L  G  G  V  L  D  Y  S  S  L  G  G  A      183
 P  S  P  P  S  M  S  W  G  A  S  Q  T  T  V  P  W  A  E  L

GCAACAGGCTCACTGCCCACCACTGCAGTAGTAGCGGCGGCAGCGGGAACAGCGAAGATC
A  T  G  S  L  P  T  T  A  V  V  A  A  A  A  G  T  A  K  I       203
 Q  Q  A  H  C  P  P  L  Q  *  *        Cfs-construct

GGCAAGGGAAGCAACTCCGGCGGAAGCTTTGATATGGGCAGGACACCAATATCGACGCAC
G  K  G  S  N  S  G  G  S  F  D  M  G  R  T  P  I  S  T  H       223
                                                Intron 1
                         NT-Box                    ↓
GGCAACAACAGCTGGGGCGGCTACGGTGGTCGTTTGCAGTTCTTTAAAGATGGCAAATTC
G  N  N  S  W  G  G  Y  G  G  R  L  Q  F  F  K  D  G  K  F       243

NT-Box
ATATTGGAACTGGCGCGGTCCAAGGATGGCGATAAAAGCGGCTGGGTTTCGGTCACG...
I  L  E  L  A  R  S  K  D  G  D  K  S  G  W  V  S  V  T  .       263
```

Supplementary Figure S1

DNA and protein sequence of the Hairless 5' region is shown. The *Kpn* I site denotes the 5' end of all the constructs. Protein sequence is numbered, starting with Met_1. The NT-Box which mediates protein contacts to Su(H) is highlighted in orange; conserved residues are shown in bold, the ones directly contacting Su(H) are shown in red. The encoding sequence is split by Intron 1, indicated by a small arrow.

The three H start codons M1 (Met_1), M2 (Met_{18}) and M3 (Met_{148}) are highlighted in bold blue. M1 can only be used if Intron 0 (light grey) is spliced; the splice donor and acceptor as well as Stop codons within the intron are bold. M3 is used by internal ribosome entry; the presumptive IRES sequences are boxed.

Primers M1V, M2V and M3V (turquoise) were used for *in vitro* mutagenesis of the Start codons to be replaced by Valine codons (GTG, bold purple). Note that mutagenesis was performed on cDNA not containing Intron 0. In the Cfs frame shift construct, a seven base pair deletion around the *Afl* II restriction site (bold, underlined) resulted in a frame shift at position 111 as depicted (grey, italics), ending translation prematurely at the novel codon 190. Hence, translation of H^{p120} can occur only by use of the IRES.

Supplementary Figure S2 **Survival rate of hemizygotes**

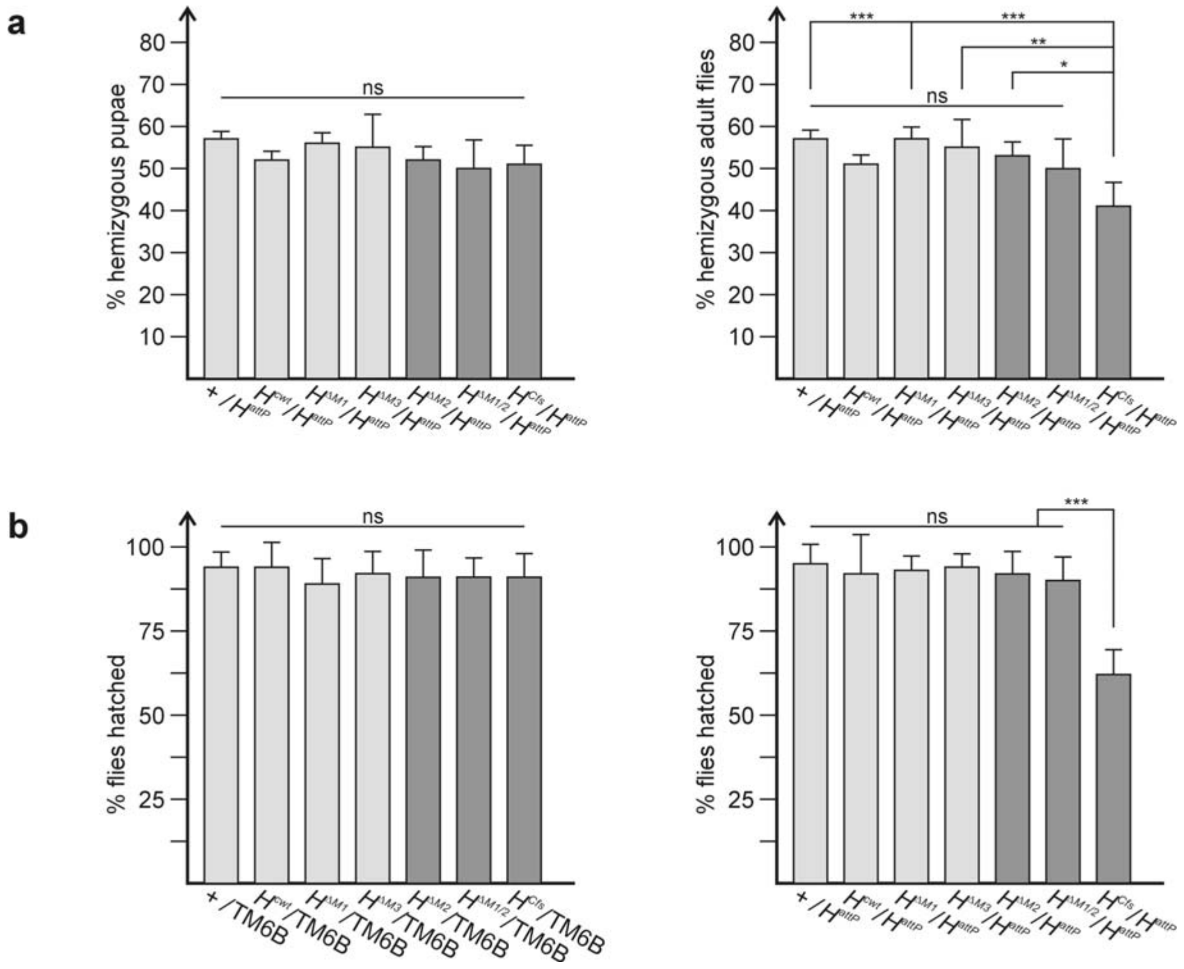

Survival rate of hemizygotes was determined by crossing the given homozygous *H** allele to the heterozygous *H* null allele H^{attP} / TM6B. Oregon 1 served as wild type control (+). Survival rate of hemizygous animals, i.e. the given allele *in trans* over the H^{attP} null allele, was assessed relative to balanced TM6B siblings.

(a) Left panel shows percentage of hemizygous pupae relative to balanced pupae: the expected 1:1 ratio is seen with all genotypes. Differences between genotypes are not significant (by ANOVA/Tukey-Kramer). Right panel compares numbers of hemizygous adults with their balanced siblings: here H^{Cfs} shows clear deficits.

(b) Shown is the percentage of adults that hatched from the pupal cases: whereas the balanced siblings (i.e. the heterozygotes) show no differences (left panel), H^{Cfs} flies appear in significantly lower numbers, whereas all other genotypes match the control (right panel).

Supplementary Figure S3 **Raw data, Westernblot from Figure 5**

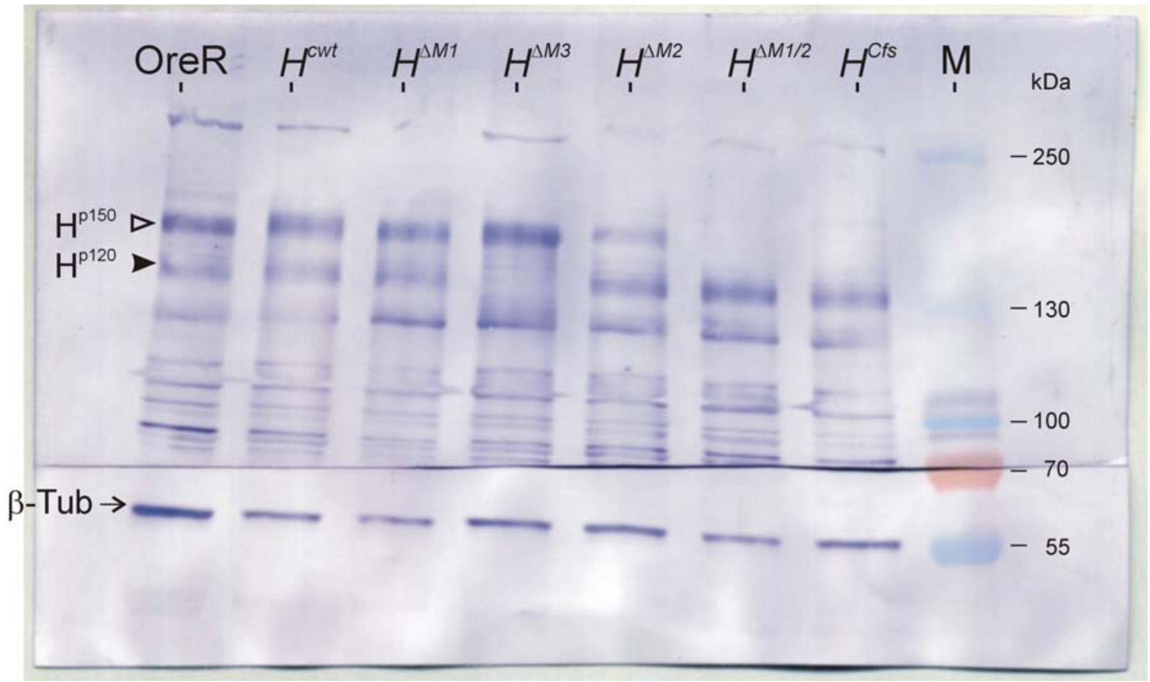

Western blot of protein extracts from 3 mg of embryos per sample of the given genotype was probed for H protein expression. A step gel was used consisting 12% (lower 2/5th) and 7.5% (upper 3/5th) polyacrylamide. The two H protein isoforms H^{p150} (open arrowhead) and H^{p120} (closed arrowhead) are indicated. Beta-Tubulin (ß-Tub) was used as loading control (arrow) on the same blot that was cut apart. Approximate size of protein standard (M) is given in kilo Dalton (kDa).
